# Comparison of the effects of photon versus carbon ion irradiation when combined with chemotherapy in vitro

**DOI:** 10.1186/1748-717X-8-260

**Published:** 2013-11-06

**Authors:** Fabian Schlaich, Stephan Brons, Thomas Haberer, Jürgen Debus, Stephanie E Combs, Klaus-Josef Weber

**Affiliations:** 1 Department of Radiation Oncology, University Hospital Heidelberg, Im Neuenheimer Feld 400, Heidelberg 69120, Germany; 2 Heidelberg Ion Therapy Centre (HIT), Im Neuenheimer Feld 450, Heidelberg 69120, Germany

**Keywords:** Human tumour cells, Carbon ion radiotherapy, Chemotherapy, Clonogenic survival

## Abstract

**Background:**

Characterization of combination effects of chemotherapy drugs with carbon ions in comparison to photons in vitro.

**Methods:**

The human colon adenocarcinoma cell line WiDr was tested for combinations with camptothecin, cisplatin, gemcitabine and paclitaxel. In addition three other human tumour cell lines (A549: lung, LN-229: glioblastoma, PANC-1: pancreas) were tested for the combination with camptothecin. Cells were irradiated with photon doses of 2, 4, 6 and 8 Gy or carbon ion doses of 0.5, 1, 2 and 3 Gy. Cell survival was assessed using the clonogenic growth assay. Treatment dependent changes in cell cycle distribution (up to 12 hours post-treatment) were measured by FACS analysis after propidium-iodide staining. Apoptosis was monitored for up to 36 hours post-treatment by Nicoletti-assay (with qualitative verification using DAPI staining).

**Results:**

All cell lines exhibited the well-known increase of killing efficacy per unit dose of carbon ion exposure, with relative biological efficiencies at 10% survival (RBE_10_) ranging from 2.3 to 3.7 for the different cell lines. In combination with chemotherapy additive toxicity was the prevailing effect. Only in combination with gemcitabine or cisplatin (WiDr) or camptothecin (all cell lines) the photon sensitivity was slightly enhanced, whereas purely independent toxicities were found with the carbon ion irradiation, in all cases. Radiation-induced cell cycle changes displayed the generally observed dose-dependent G2-arrest with little effect on S-phase fraction for all cell lines for photons and for carbon ions. Only paclitaxel showed a significant induction of apoptosis in WiDr cell line but independent of the used radiation quality.

**Conclusions:**

Combined effects of different chemotherapeutics with photons or with carbon ions do neither display qualitative nor substantial quantitative differences. Small radiosensitizing effects, when observed with photons are decreased with carbon ions. The data support the idea that a radiochemotherapy with common drugs and carbon ion irradiation might be as feasible as respective photon-based protocols. The present data serve as an important radiobiological basis for further combination experiments, as well as clinical studies on combination treatments.

## Background

Today the combined radiochemotherapy with photons is a well-established part of interdisciplinary tumour treatment for many tumour entities [1]. The molecular and cellular fundamentals as well as the clinical implications are well known for combined radiochemotherapy with photons and are subjects to further preclinical and clinical investigations. Heavy ion radiotherapy offers distinct biological and physical characteristics for a precise tumour treatment with simultaneous protection of normal tissues: the inverted dose profile with low dose deposition in the entry channel and high dose deposition in the so called Bragg peak leads to normal tissue protection and reduction of the integral dose to the patient. Heavy ions also produce severe radiation damage within the beam track leading to a higher relative biological effectiveness (RBE) for heavy ions in comparison to photons [2-4]. Most likely the higher efficiency in cell-killing of heavy ions is based on complex and/or densely spaced DNA double-strand breaks which are difficult to repair for the cells [5,6]. The clinical use of heavy ion radiotherapy is subject of several clinical investigations [7,8].

Combined radiochemotherapy with photons is well-established in the clinical practice based on various preclinical and clinical investigations, however, there is only little data for the combination of chemotherapy with heavy ions. Due to the different radiobiological effects of heavy ions especially to the impact on cell cycle control, the known combination effects from photons in combination with chemotherapies of different working mechanisms may differ from the combination effects from heavy ions. In vitro investigations by Kitabayashi and colleagues evaluated carbon ion irradiation in combination with different chemotherapies in oesophageal squamous cell carcinoma cells. They found additive effects for all combinations except for docetaxel which showed synergistic combination effects [9]. Previous in vitro investigations for combined radiochemotherapy with carbon ions in glioblastoma cells showed an RBE ranging between 3.3 and 3.9 depending on survival level and dose. Combination with chemotherapies of different mechanisms of action demonstrated additive effects with dominant effects produced by paclitaxel and camptothecin [10].

An expansion of the clinical implication of heavy ion radiotherapy must include the combination with chemotherapies. The ambition of the present analysis is to evaluate the combination effects of carbon ion irradiation when combined with different chemotherapies in a human adenocarcinoma cell line which was previously used in our lab to support radiochemotherapy studies. In addition, since the drug camptothecin revealed some indications of radiosensitization, three other cell lines (glioblastoma, lung, pancreas) were tested for combination with camptothecin as well.

## Methods

### Cell culture

The human tumour cell lines A549 (lung), LN-229 (glioblastoma), PANC-1 (pancreas) and WiDr (colon) were obtained from the American Type Culture Collection (ATCC, Manassas, VA, USA). The cells were cultured in DMEM supplemented with 10% foetal calf serum (FCS), respectively in RPMI 1640 supplemented with 10% foetal calf serum (PANC-1). They were maintained in culture at 37°C in humidified air with 6% CO_2_.

### Photon irradiation

Photon irradiation was performed as single dose exposure to photon doses of 2, 4, 6 and 8 Gy at room temperature. Photons were delivered by a biological irradiator (Precision X-Ray, North Branford, CT, USA). Photons were delivered at 320 kV and 12.5 mA with an averaged dose rate of 1 Gy per minute.

### Carbon ion irradiation

Carbon ion irradiation was performed at the Heidelberg Ion Therapy Centre (HIT) using a horizontal beamline with rasterscanning technique. To obtain clinically relevant parameters in cell irradiation the dose was delivered as an extended Bragg peak with 103 keV/μm (dose averaged LET). The cell monolayers were irradiated in the middle of the extended Bragg peak adjusted by using a 3 cm acrylic shield. Single carbon ion doses of 0.5, 1, 2 and 3 Gy were applied with an averaged dose rate of 0.5 Gy per minute.

### Chemotherapeutic treatment

All cell lines were treated with chemotherapy concentrations at medium toxicity level (approximately 50% clonogenic survival) for 4 hours followed by a medium change prior to irradiation. This is an exposure scheme firmly established in our laboratory to limit drug exposure time because active clearance mechanisms as present in the whole organism are absent in the culture flask. Of course a direct simulation of the clinical situation is not possible. WiDr cell line was tested for camptothecin (Enzo Life Sciences GmbH, Lörrach, Germany), cisplatin, gemcitabine and paclitaxel (all obtained from the pharmacy of University Hospital Heidelberg), all other cell lines were tested for combination with camptothecin.

For all substances, dose–response-relationships were generated for single-agent treatment. Following drug concentrations were chosen for combination experiments: camptothecin at 80 nM (A549), 75 nM (LN-229), 25 nM (PANC-1) and 100 nM (WiDr), cisplatin at 2 μM, gemcitabine at 70 nM and paclitaxel at 25 nM.

### Clonogenic assay

The effects of single and double treatment on cell survival were evaluated using the clonogenic growth assay representing the radiobiological gold standard. Due to the fact that the final cell death may occur only after additional cell divisions the clonogenic survival is an important marker of cell survival after treatment with ionizing irradiation and antitumour reagents. All cell lines were grown under standard conditions. For analysing the effects of photon or carbon ion irradiation alone or in combination with chemotherapy on clonogenic survival, increasing numbers of cells were plated in 25 cm^2^ flasks (Sarstedt, Nümbrecht, Germany). After an attachment period cells were exposed to specific treatment and kept in the incubator for 10–14 days after which they were stained with crystal violet (Serva Electrophoresis GmbH, Heidelberg, Germany). Colonies were counted by microscopic inspection and only colonies with at least 50 cells were counted. Plating efficiencies and survival fractions were calculated. Each experiment was performed in triplicates and was repeated at 3 different days. Mean values and standard deviations were only calculated from independent experiments. Analysis of statistical significance was performed using the t-test. In combination experiments an approach was invoked utilising the criteria of additivity described by Steel and Peckham [11]. The following definitions were used for the description of drug-radiation interactions: independent toxicity denotes the effect of the combination of two agents where total effect is a product of the effects of single agents. This mechanism needs no interaction between drugs and radiation, or their biological effects. The term additivity and supra-additivity according to Steel and Peckham (see above) is defined for a range of combination toxicities. This range is restricted by the dose–response curve of radiation and the isoeffect curve of radiation on the effect level of chemotherapy, alone. Dose–response curves located within this range represent additive effects, when exceeding this range they describe supra-additive effects (or synergism).

### Cell cycle analysis and monitoring of apoptosis

Treatment dependent changes in cell cycle distribution were analysed at 4 different time points: at the beginning of chemotherapy treatment, directly post-irradiation, 8 and 12 hours post-irradiation. After trypsinization the cells were washed and fixed in 70% ethanol. Fixed cells were centrifuged, washed and incubated in RNAse and propidium iodide prior to measurement of DNA-content using a FACScan flow cytometer (Becton Dickinson, Heidelberg, Germany) [12]. Each experiment was repeated at 3 different days. Mean values and standard deviations were only calculated from independent experiments.

Apoptosis was monitored at 12, 24 and 36 hours post-irradiation by Nicoletti-assay [13]. Trypsinized cells were washed and fixed in 70% ethanol. Fixed cells were centrifuged, washed and incubated in a hypotonic fluorochrome solution (PI 50 μg/ml in 0.1% sodium citrate plus 0.1% Triton X-100) in the dark overnight before flow-cytometric analysis. Each experiment was repeated at 3 different days. Mean values and standard deviations were only calculated from independent experiments. Qualitative verification was performed using DAPI staining and microscopic inspection.

## Results

### Clonogenic survival and determination of RBE

The clonogenic survival was calculated for all used cell lines for increasing doses of photon or carbon ion irradiation. All cell lines exhibited the well-known increase of killing efficacy per unit dose of carbon ion exposure leading to a steeper dose–response-relationship for carbon ions compared to photons. The strongest effect was seen for A549 cell line.

From these values we calculated the relative biological efficiencies at 10% survival level for each cell line (Table 1). These RBE_10_ values range from 2.3 to 3.7 depending on the cell line.

**Table 1:** **RBE**_**10 **_**values for different cell lines**

	**WiDr**	**A549**	**LN-229**	**PANC-1**
**no drug**	**3.1 ± 0.7**	**3.6 ± 0.8**	**2.7 ± 0.5**	**2.4 ± 0.4**
**camptothecin**	**2.6 ± 0.3**	**2.8 ± 0.5**	**2.5 ± 1.1**	**2.2 ± 0.2**
**cisplatin**	**2.6 ± 0.01**	**-**	**-**	**-**
**gemcitabine**	**3.1 ± 0.1**	**-**	**-**	**-**
**paclitaxel**	**3.2 ± 0.5**	**-**	**-**	**-**

Mean values of relative biological efficiencies at 10% survival (RBE_10_) (plus standard deviations) for irradiation alone or in combination with chemotherapy drugs.

### Evaluation of cytotoxic effect of combination therapies

**Figure 1:**
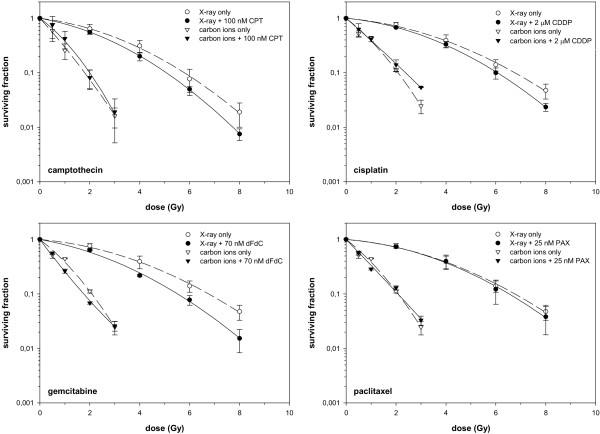
**Combination experiments of 4 chemotherapeutic drugs in WiDr cell line, as indicated.** Clonogenic radiation survival of log-phase WiDr cells. Cells were irradiated with photons or carbon ions only or directly after a 4-hour treatment with camptothecin, cisplatin, gemcitabine or paclitaxel and a following medium change. Data points are mean values (plus standard deviations) from three independent experiments. The survival curves were derived from a fit of the linear-quadratic survival expression to the data.

Combination experiments of 4 chemotherapeutic drugs camptothecin, cisplatin, gemcitabine and paclitaxel with both radiation qualities for WiDr cell line were performed (Figure 1). For generation of the survival curves within each independent experiment, PE-values measured in triplicate for each treatment condition and the averaged values were normalized to the respective number of untreated sample. Respective PE-values in the combination experiments were normalized to a drug control yielding surviving fractions displayed in the graphical representation. Slightly enhanced photon sensitivity was found in combination with camptothecin, cisplatin and gemcitabine, whereas all other combinations showed only independent toxicities.

**Figure 2:**
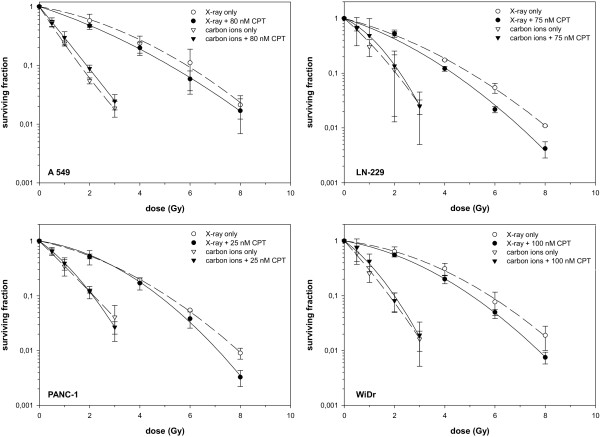
**Combination experiments of camptothecin in 4 different cell lines, as indicated.** Clonogenic radiation survival of 4 different log-phase tumour cell lines. Cells were irradiated with photons or carbon ions only or directly after a 4-hour treatment with camptothecin and a following medium change. Data points are mean values (plus standard deviations) from three independent experiments. The survival curves were derived from a fit of the linear-quadratic survival expression to the data. Note, that the WiDr panel is the same as in Figure 1 for camptothecin but now in the changed context.

For generation of the survival curves of combination experiments of camptothecin for all cell lines the same procedure was performed (Figure 2). Results showed slightly enhanced photon sensitivity for all cell lines in combination with camptothecin, whereas purely independent toxicities were found with carbon ions leading to lower RBE_10_-values for combination treatment (Table 1).

### Cell cycle distribution after irradiation and chemotherapy treatment

**Figure 3:**
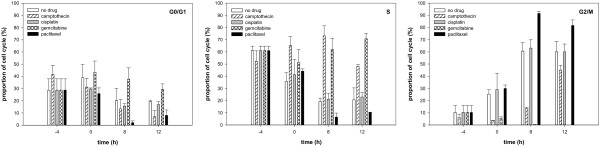
**Cell cycle distribution (as indicated) of WiDr cells treated with 8 Gy photons and 4 different chemotherapy drugs.** Change of cell cycle distribution of log-phase WiDr cells. After a 4-hour treatment with camptothecin, cisplatin, gemcitabine or paclitaxel and a following medium change cells were irradiated with 8 Gy photons. Cell cycle distribution was measured by flow cytometry after propidium iodide staining up to 12 hours post-irradiation. Data points represent mean values (plus standard deviations) from three independent measurements.

**Figure 4:**
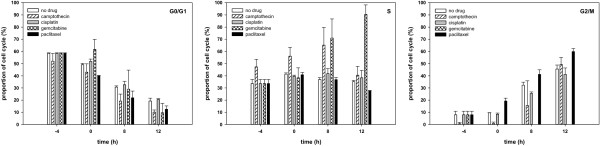
**Cell cycle distribution (as indicated) of WiDr cells treated with 3 Gy carbon ions and 4 different chemotherapy drugs.** Change of cell cycle distribution of log-phase WiDr cells. After a 4-hour treatment with camptothecin, cisplatin, gemcitabine or paclitaxel and a following medium change cells were irradiated with 3 Gy carbon ions. Cell cycle distribution was measured by flow cytometry after propidium iodide staining up to 12 hours post-irradiation. Data points represent mean values (plus standard deviations) from three independent measurements.

All cell lines showed the prevailing dose-dependent G2-arrest after photon and carbon ion irradiation while the maximum for carbon ions was not reached in the analysed time. For all cell lines a cell cycle delay after carbon ion irradiation was observed also in combination therapy. In combination with camptothecin a drug-specific halt in S-phase up to 8 hours post-irradiation with transition into the G2-arrest was seen for all cell lines. Combination with gemcitabine showed a drug-specific halt in S-phase for more than 12 hours post-irradiation with delayed occurrence of G2-arrest for WiDr cell line. In combination with cisplatin no specific influence on cell cycle distribution was visible for WiDr cell line. Paclitaxel showed a stronger increase in G2/M-phase fraction in combination compared to irradiation alone (Figures 3, 4).

### Treatment specific apoptosis after irradiation and chemotherapy treatment

**Figure 5:**
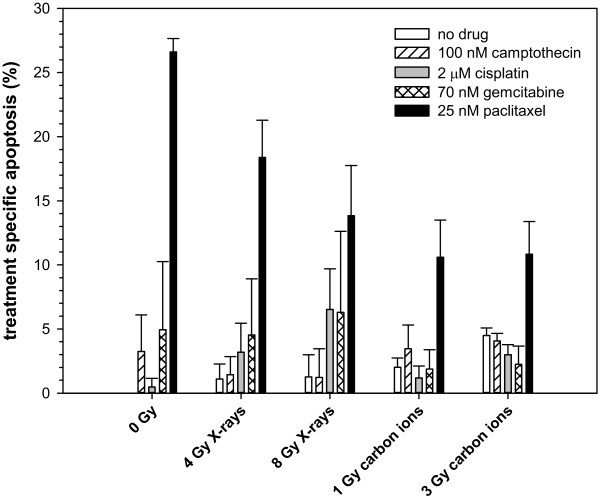
**Treatment specific apoptosis of WiDr cells at 36 hours post-irradiation.** Cells were treated for 4 hours with camptothecin, cisplatin, gemcitabine or paclitaxel followed by a medium change and irradiation with 4 or 8 Gy photons or 1 or 3 Gy carbon ions. Apoptosis was measured by flow cytometry using Nicoletti-assay. Data points represent mean values (plus standard deviations) from three independent measurements.

All cell lines showed no significant induction of apoptosis neither for irradiation alone nor for combination with camptothecin. For WiDr cell line only paclitaxel showed a significant induction of apoptosis but independent of the used radiation quality (Figure 5).

## Discussion

Our experiments evaluated the combination of different chemotherapeutic substances with photon and carbon ion irradiation with respect to cell survival in different human tumour cell lines. A slightly enhanced photon sensitivity was found for the combination with camptothecin for all cell lines and for cisplatin and gemcitabine in WiDr cell line. All other combinations showed only independent toxicities.

Until now, few data is available on the combination of chemotherapy and high-LET particle beams, such as carbon ion radiotherapy.

Camptothecin acts by binding to and stabilizing of the covalent binary complex DNA-topoisomerase I leading to inhibition of religation of the cleaved strand and DNA double-strand-breaks [14]. Today, two camptothecin analogues are in clinical use: topotecan and irinotecan [14,15]. Djuzenova et al. [16] investigated the combined treatment with camptothecin and ionizing radiation in five glioma cell lines. They found radiosensitization after camptothecin pretreatment only in two of five cell lines. Rave-Fränk et al. [17] showed radiosensitization for photon irradiation in WiDr cell line after 24 h pretreatment with topotecan. These results are conclusive to the effects we found.

The antitumour activity of cisplatin is attributed to the kinetics of its chloride ligand displacement reactions leading to DNA crosslinking activities. DNA crosslinks inhibit replication, transcription and other nuclear functions and arrest cancer cell proliferation and tumour growth. A number of additional properties of cisplatin are emerging, including activation of signal transduction pathways leading to apoptosis [18]. Today cisplatin, usually in combination with other drugs, is being used as first-line chemotherapy against many cancers, as well as as second-line treatment [19]. In vitro studies for combination treatment with cisplatin and ionizing radiation showed differential results. Flentje et al. [20] observed additive effects for four human cell lines independent of simultaneous or sequential cisplatin exposure. Geldof et al. [21,22] found supra-additive treatment effects of cisplatin with radiotherapy in prostate cancer cell lines. Zhang et al. [23] investigated concurrent cetuximab, cisplatin and radiation in seven HNSCC cell lines. Only one cell line showed supra-additive effects for the combination of cisplatin and radiation, all others showed only additive effects. Our experiments showed also additive effects for combination of cisplatin and photon irradiation but only independent toxicities for the combination with carbon ion irradiation.

Gemcitabine is converted intracellularly to the active metabolites difluorodeoxycytidine di- and triphosphate (dFdCDP and dFdCTP). dFdCDP inhibits ribonucleotide reductase, thereby decreasing the deoxynucleotide pool available for DNA synthesis. dFdCTP is incorporated into DNA, resulting in DNA strand termination and apoptosis [24]. Gemcitabine has wide a spectrum of activity against solid tumours and is currently under investigation in many clinical trials. Latz et al. [25] could show a supra-additive effect for the combination of gemcitabine and photon irradiation in WiDr cells when irradiation was performed at the end of gemcitabine exposure (2 h). For all other points of time only additive effects were observed. They could also show an S-phase-specific radiosensitization by gemcitabine for serum stimulated WiDr cells. These results were confirmed by Harrabi et al., which could also show an S-phase-specific radiosensitization by gemcitabine for synchronized WiDr cells when irradiated with carbon ions whereas log-phase cells only showed additive effects [unpublished data; personal communication by SB Harrabi]. Our experiments also showed slightly enhanced photon sensitivity in log-phase WiDr cells when pretreated with gemcitabine (4 h). When irradiated with carbon ions these cells showed only independent toxicities. These differences between photon and carbon ion irradiation might be explained by the fact that the effectiveness of carbon ions is less cell-cycle-dependent.

Paclitaxel binds to β-tubulin, stabilizes the microtubule and thereby inhibits depolymerization. This disrupts the normal dynamic reorganization of the microtubule network required for mitosis and cell proliferation and results in the arrest of cells in the G_2_M-phase at passing from metaphase to anaphase. Drug-blocked cells may eventually exit mitosis, often aberrantly. Mitotically blocked or mitotically slowed cells eventually die by apoptosis [26-28]. The G_2_M-phase is the most radiosensitive phase of the cell cycle and thus paclitaxel should act as a potent radiosensitizer. Tishler et al. [29] could show a more than additive effect after 24 hours of 10 nM paclitaxel exposure and subsequent photon irradiation in G18 cell line. At this point of time nearly 100% of cells were in G_2_M-phase. The same effect was demonstrated by Wenz et al. [30]: photon irradiation given 6 hours after 0.3 μM paclitaxel for 2 hours showed a considerable increase in radiosensitivity. When irradiation was given 4 hours after the beginning of paclitaxel exposure radiosensitivity was slightly reduced. In conclusion maximal radiosensitization of paclitaxel was seen when irradiation was given at the time of maximal G_2_M accumulation of cells. They also showed that p53wt TK6 cells were permanently blocked in G_2_M whereas p53mut WTK1 cells were only transiently blocked, suggesting a function of p53 in the response to paclitaxel. In our experiments WiDr cells showed only independent toxicities regardless of the used radiation quality. WiDr cells were irradiated after 4 hours incubation with 25 nM paclitaxel. At this point of time the G_2_M fraction was about 20% and about 40% of cells were in S-phase. This could be a possible explanation for the absence of radiosensitization of paclitaxel. Another hypothesis for the absence of radiosensitization when combined with carbon ions is a possible reduction of the cross-section area at maximal G_2_M accumulation by beginning chromatin condensation in the early metaphase. For evaluation of this hypothesis further experiments are required.

WiDr cells showed a significant induction of apoptosis after treatment with paclitaxel independent of the used radiation quality, suggesting that apoptosis is induced by paclitaxel. As WiDr cell line is p53 mutated the induction of apoptosis by paclitaxel must be independent of p53.

A recently published study by Combs et al. [10] evaluated the combination of carbon ion radiotherapy with chemotherapy in U87 cell line and showed similar results compared to ours.

In our experiments a cell cycle delay was seen after carbon ion irradiation alone and in combination with different chemotherapeutics. Scholz et al. [31] could show a prolongation of delays with increasing LET while a large fraction of cells remained in S or G_2_M phase for up to 48 h or longer after irradiation. Already early investigations of Lücke-Huhle et al. [32] in V79 cell line revealed high-LET particle beams to be much more effective in causing a G_2_M block than low-LET radiation, and the RBE values for G_2_M block were much higher than the RBEs for survival. This is consistent with our findings although the maximum G_2_M accumulation after carbon ion irradiation was not reported in the measured time points.

This manuscript presents an extensive evaluation of the combination effects of carbon ions and chemotherapy drugs of different mechanisms of action. Therefore the present work represents essential radiobiological information for subsequent preclinical as well as clinical applications, leading to the conclusion that when such substances are combined with carbon ions no specific reductions in dosing with respect to cytotoxicity in the radiation area must be undertaken compared to combined treatments with photons.

## Conclusions

Combined effects of different chemotherapeutics with photons or with carbon ions with respect to in vitro survival do neither display qualitative nor substantial quantitative differences. Small increased effects above independent toxicities, when observed with photons are decreased with carbon ions. The data support the idea that a radiochemotherapy with common drugs and carbon ion irradiation might be as feasible as respective photon-based protocols. The present data serve as an important radiobiological basis for further combination experiments, as well as clinical studies on combination treatments.

## Competing interests

The authors declare that they have no competing interests.

## Authors’ contributions

FS, JD, SEC and KJW designed and planned the experiments. FS performed the experiments. SB and TH performed radiation therapy treatment planning and application. FS and KJW wrote the manuscript and made the graphical illustrations. All co-authors approved the manuscript.
